# Why twin studies are important for health span science research: the case of maltreatment of aging adults

**DOI:** 10.1186/s12877-022-03440-6

**Published:** 2022-12-08

**Authors:** Brian B. Boutwell, Chelsey S. Narvey, Jesse J. Helton, Alex R. Piquero

**Affiliations:** 1 The University of Mississippi, University of Mississippi Medical Center, 84 Dormitory Row West, P.O. Box 1848, University, MS 38677 USA; 2 Department of Criminal Justice and Criminology, Sam Houston State University, 1905 University Ave, Huntsville, TX 77341 USA; 3 Saint Louis University, 3550 Lindell BLVD, Saint Louis, MO 63103 USA; 4 Department of Sociology and Criminology, University of Miami, 5202 University Drive Merrick Building, Coral Gables, FL 33124 USA

**Keywords:** Quantitative genetics, Health span, Quasi-experimental, Causality, Twins

## Abstract

Average life expectancies have lengthened across human history. As a result, there is an increased need to care for a greater number of individuals experiencing common age-related declines in health. This has helped to spur a rapidly increasing focus on understanding “health span”, the portion of the life-course spent functionally healthy. Yet to penetrate the science of health span, however, is a topic which seems fundamental to the ability to age in functional and healthy ways, and has received considerable attention in other fields. As more of the population ages, the risk of exposure to abuse and neglect among older citizens not only rises, but can manifest as both *cause* and *effect* of declining health span. Among our goals here is to make a case for including this subject among the other central components of health span science. In so doing, we also outline reasons why quantitative genetic designs using samples of twins can be a versatile tool for improving causal inference when studying maltreatment among older persons specifically, but also on a range of other health span topics in general.

## Background

Human life expectancies have lengthened over time [1–3]. Accompanying this trend has been a growing interest in the study of aging, driven in no small part by the fact that with each decade lived, the odds of developing certain medical conditions rise considerably [2, 4, 5]. These common diseases of aging have the capacity to dramatically erode functional health, thus the field of health span emerged in recent decades [4, 6]. Moving beyond questions of how to increase the number of years alive, health span broadens its thinking to include how to maximize quality of life for as long as possible.

The amount of scholarly work conducted under the auspices of health span science is rapidly expanding [4]. Medical outcomes like cancer, diabetes and cardiovascular disease understandably attract tremendous amounts of scrutiny, for the obvious reason of that they become increasingly likely as one ages (see [4]). We suspect, too, that as this field continues its expansion, scholars will increasingly focus on psychological and behavioral outcomes; variables often linked to medical conditions, and which have the capacity to impact quality of life directly and indirectly. Part of our purpose here, indeed, is to call attention to an important issue linked to a longer-lived populace, but which may not leap immediately to mind. As the number of older adults rises, so too will the likelihood that some number of them will be physically or psychologically abused and neglected (see also [5]).

Maltreatment of older persons is not a medical disease, per se, but it does represent an important correlate of health span. The necessity of adult care that often comes with age can intertwine itself with quality of life in numerous and sometimes unexpected ways. Further complicating the issue is that quality of care might impact health span directly, and it may also be impacted *by* health span variables. Certain age-related diseases necessitate the need for increased provisioning of care (e.g., dementia), which by extension can increase risk of exposure to abuse or neglect [5, 7]. Maltreatment can exist as a dependent variable, independent variable, and a mediating variable in relation to health span outcomes. Adding to the challenge is the fact that exposure to maltreatment falls into a category of “treatment” variables closed to randomization. This is not by definition a weakness. It is a reality, though, one that entails certain challenges when the goal is to understand causal processes [8–10].

These are the primary considerations that animate our discussion. We argue that the study of abuse and neglect of older adults should be added to the core research areas in the health span science. In doing so, we present a strategy for dealing with the methodological hurdles that exist for studying variables which cannot be analyzed experimentally. Among the most important themes of the discussion, in fact, is that the methodological difficulties we focus on are not isolated to our particular topic. Fortunately, the strategies on offer to address them are *general* and can find a home in practically any area of health span scholarship.

### The shifting face of aging research: lifespan and health span

Research on health span began exploding some years back, and interestingly one of our key focal points started becoming apparent early on. Many of key topics in the newly emerging field would benefit from using an interdisciplinary developmental framework, coupled with longitudinal designs tracking participants across large portions of the life-course (see [4]). Also apparent was the fact that the topics themselves would often prove difficult or impossible to test experimentally [4]. The brief survey of health span studies we include here is not intended to be systematic or exhaustive. Instead, it is intended to reflect a reality encountered by health span researchers and to demonstrate how certain types of data and analytical strategies can help to improve causal inference abilities when randomized manipulation of treatments is not an option. We begin with heart disease, as it falls directly into this category.

Heart disease is a looming public health threat with considerable prevalence and a risk profile that increases sharply with age [11, 12]. Kulminski and colleagues [13] provide a useful example of how complex topics like heart disease can be creatively studied, despite an inability to always perform a randomized trial. To better clarify why the topic calls for interdisciplinary creativity at all, it’s important to consider a few points. It has been understood for some time that there is a role for genetic influences to play in the etiology of the condition [14]. Moreover, given the life-course progression of heart disease, one of the more useful approaches for studying it involves longitudinal data, and specifically longitudinal data which contain both genetic and environmental variables.

Such data exists, and Kulminski and colleagues [13] made use of it, noting among several interesting results that the effects of trait relevant genes on disease outcomes can vary with age. Moreover, these age-related genetic effects might be moderated by certain environmental exposures [13]. In particular, they evaluated the possible effects of lipid-related genes, aging-related processes, and changing environments on health span using data from the original Framingham Heart Study (FHS) and the FHS Offspring cohorts in which subjects were followed for approximately 60 years. Additional analyses suggested that certain genetic variants—*APOE* e4 allele and *APOB* CC—seemed to also have sex-specific effects on the development of cardiovascular disease at different ages and in different environments.

Focusing on cancer and diabetes, among other key diseases, Sebastiani and colleagues [15] examined subjects in the Long Life Family Study (LLFS) and New England Centenarian Study (NECS) along with controls without family histories of pronounced longevity. Using Bayesian survival analysis, the authors estimated age of onset of disease and years of disease-free survival. Their findings suggested that individuals in the LLFS had significantly lower risks for several illnesses including cancer and diabetes. Importantly, the age at which a significant portion of the sample (at least 20%) developed one of the focal diseases was roughly a decade later than controls, highlighting not only a longer life span, but also a longer health span.

These are but two studies among many others that we might have reviewed. We selected them, not because they represent the pinnacle of research, but because they seem representative both of topics emphasized in the health span literature, as well useful data sources when longitudinal samples are needed, and experiments are not an option. We could have included Alzheimer’s disease here, too, as it is among the most widely known and feared diseases of aging [16]. We discuss it at a later point, in fact. The tendency to focus heavily on the most prominent diseases of aging is entirely defensible, as the impact is so diffuse in the population. We contend that expanding the variables studied under the auspices of health span science warrants strong consideration, yet it also requires careful thought about the research strategies employed. This matters, because despite the ability to employ complex statistical analyses with longitudinal data, non-experimental data remain vulnerable to specific problems, two of which we discuss below [8].

### Two concerns for health span: confounding and selection bias

Observational data is used widely across the health sciences already, and this is not a criticism, simply a fact. Observational data, especially from large prospective and representative cohorts, continues to be essential for addressing a variety of questions surrounding health and wellness. That said, trade-offs always exist when deciding on a particular research design. Depending on whether the design is experimental or non-experimental, one key trade-off involves the ease with which one can manage selection bias and confounding [12]. Depending on the data, there are options and many of them offer excellent causal inference capabilities when certain assumptions are met [17, 18].

We focus particularly on quantitative genetic approaches, a broad term that references research designs applied to data containing multiple subjects from the same family [19, 20]. Quite often this involves using twin and non-twin siblings of varying relatedness (e.g., monozygotic twins, dizygotic twins, and siblings (see [14, 20]). To understand the utility of twin and other family based designs, we can start by noting the broad consensus which as emerged from decades of such work, suggesting that *both* genetic and environmental factors are typically involved in accounting for variance across numerous socially, psychologically, and medically-relevant variables [14]. Though perhaps not immediately obvious, the most relevant point here involves certain methodological implications. Properly executed randomized experiments account for genetic and environmental factors which might impact causal inference [10]. Such tasks are more difficult for observational studies [10, 20]. Much of the usefulness of quantitative genetic designs, then, concerns their ability to act as quasi-experimental tools in non-experimental data [10, 21].

Numerous factors can conspire to limit the use of experimental designs, some of which are pragmatic in nature and do not necessarily concern the ethics of certain treatment exposures. A well-known illustration of this is TAME (Targeting Aging with Metformin) [22]. The anti-diabetic drug metformin is among the most widely prescribed and generally safe drugs in the world, satisfying most of the larger concerns about randomly assigning individuals to its use [6, 22]. Motivating TAME is nascent evidence of numerous beneficial effects, beyond glucose control, which might accompany metformin use in diabetics and non-diabetics alike [22, 23]. There is little reason to dispute the argument that large, multi-site clinical trials are important. But, they require massive investment in the form of money, coordination of research personnel, and time. Funding streams are limited in the best of times, but they can quickly become even more tightly constricted depending on historical context, such as the arrival of a global health crisis. The most recent in memory, of course, involving the need to expedite treatment and vaccination development for the COVID-19 pandemic.

All of these practical considerations conspire to make multi-site clinical trials arguably more rare than health span researchers might prefer. Helping to keep research productivity moving forward, luckily, is the relatively large number of well-powered observational databases. At a minimum, these resources provide opportunity to correlate relevant variables with a range of health span related outcomes, often longitudinally across years and even decades of the life-course. Helping matters more is the fact that other than funding required to initiate and complete a given study, the typically become free and easily accessible to any interested researcher. Despite these, and other estimable qualities, observational data harbor the shortcomings of correlational designs that we have been eluding to [10, 21, 24]. Our central contention is that the application of quantitative genetic designs when possible can help to elevate observational data in terms of causal inference capabilities [2, 8].

Though we lack the space to discuss the granular details, quantitative genetic studies utilizing twins and other sibling types can control for various forms of confounding, permitting causal inference in ways that associational designs typically cannot [9, 10]. Virtually all complex traits, as we have noted, emerge from multiple causal pathways and are heritable to some degree [14, 25]. It is this quality that underscores the relevance of twin and sibling designs. Considered through the lens of causal inference, designs including twins and siblings open the possibility of better controlling shared genetic and environmental confounding factors in ways frequently closed to other analytical strategies in observational data [10].

Some further explication here would be worth the time, in order to conceptualize why standard approaches to observational data analysis can be limited for causal inference. In the absence of experimental control, researchers employ statistical controls in an effort to “hold constant” variables considered relevant to the research question in general [10]. More specifically, the focus tends to center on variables thought to represent confounding influences in the data [26]. Reliance on statistical controls is often a necessity and indeed can be the appropriate course of action. Still, some points of caution warrant a discussion here. As a starting point for thinking carefully about this topic, Lee offered a succinct description of what is happening when one controls some variable statistically ([10]; p.375):

“*Recall that statistically controlling for a variable Z, in an attempt to determine whether X affects Y, amounts to observing the association between X and Y in a subpopulation where all members share the same value of Z. In the language of probability theory, we are ‘conditioning on’ this particular value of Z*.”

Compelling arguments concerning the causal effects of different variables can certainly be made using statistical control in regression models, for example [24, 26]. The veracity of those arguments, however, partly hinges on a thorough knowledge about the variables that must be controlled in one’s equations [10]. Additionally, a sometimes underappreciated point is that one needs to be aware of variables that should be purposely excluded from the equations, so as to avoid the introduction of new problems, such as collider bias [10, 26]. If we assume that knowledge of a particular topic will generally always be less than complete, we confront the reality that *statistical* control can often prove inferior to *experimental* control [10, 24, 26]. None of this is a reason to avoid observational data, nor does it council despondency about existing research. What it does do is advise caution when specifying a causal argument using non-experimental research designs.

When experimental control is lacking, another difficulty presents itself, that of non-random selection into an exposure variable [24]. This is not new, of course, and behavioral scientists have been thinking carefully about the issue for decades [10]. Though she was not contemplating our specific subject per se, Scarr’s ([27]; see also [28]) writings about this concern are instructive. Characterizing this problem of selection bias as gene-environment correlations (rGEs), she proposed a general framework intended to describe the ways in which humans across all ages play at least some role in shaping their own environments. Scarr was concerned with better describing how and why certain environmental experiences—be they positive or deleterious—collide with some individuals and not others. rGEs can manifest in three varieties: passive, evocative, and active [27, 28].

Passive rGEs capture situations in which biological parents—each of whom provide half of their genetic material to a child—are also involved in shaping their child’s suite of experiences. A classic example involves active parents filling a child’s environment with athletic commitments [27]. Because physical abilities are partly heritable, the child’s environment acts to reinforce traits impacted by genetic overlap shared with parents [29, 30]. Evocative rGE reflects the correlation between experiences and the temperaments and personality of an individual. Scoring high on trait extraversion, for instance, might over time play a role in creating a range of experiences for that person, that differ in certain aspects from those of someone scoring lower on that particular dimension [27]. Active rGEs reflect a tendency to “actively” seek environments conducive to our own interests and abilities. This type of self-selection is a key issue that randomization can short circuit in experiments. Individuals often opt for experiences that align with their personal preferences [8, 9]. Generally speaking Scarr’s [27] concepts form a foundation for better understanding how humans exert some degree of agency in creating their own environments as we age. In this context, they also collide with issues of health span.

It seems uncontroversial to assert that experiences at various points in life are relevant for understanding and forecasting the state of someone’s wellbeing as they age. What cannot be underscored enough, though, is that many of these experiences embody two critical qualities: 1) they are encountered non-randomly owing to individual-level differences as we just described and 2) we could potentially randomize exposure to these experiences in order to parse their causal effects. Doing so, however, would either prove unfeasible, dangerously unethical, and typically would in fact be both [24]. Intentional exposure to abuse and neglect at any age certainly qualifies as unethical. That said, we can move on to describing strategies for surmounting these methodological challenges by reviewing some of the existing work with twins. We focus on two examples in particular.

### Merging health span, adult maltreatment and twin studies: two examples

In the first example, McGue and colleagues already anticipated the foundation of our arguments about studying twins in health span and gero-sciences noting that ([2]; p.549):

“*The extent to which the discordant-twin design will be of utility in gerontology will depend on the degree to which exposure to putative aging risk factors is heritable, just as Fisher (1958) reasoned for smoking more the 50 years ago. That is, the power of the discordant-twin design is that it controls for potential genetic (and also shared environmental) confounding, and without heritability there can be no genetic confounding.*”

The partial heritability of many complex human traits is a finding which has replicated consistently across fifty years of research, to the point that it is no longer surprising per se to discover that some trait has a non-zero heritability estimate [14, 31, 32]. From our perspective, the point of using twin data in this case is not necessarily to calculate heritability estimates, but to capitalize on specific modeling techniques which can strengthen causal inference abilities in observational data [8–10, 33].

A common approach, for instance, involves the estimation of fixed effects regression models using twin pairs [33]. To illustrate, McGue and colleagues [2] analyzed data drawn from the Longitudinal Study of Aging Danish Twins (LSADT) in order examine the connection of alcohol consumption and cognition with age. The sample included dizygotic (DZ; n = 597 same sex siblings) and monozygotic (MZ; n = 412) twins. As a reminder, DZ twins share 50 percent of their distinguishing genetic material, MZ pairs are identical in this regard. A key part of the logic in these studies, then, is that when differences on some variable emerge—particularly for MZ twins—they should stem primarily from exposure to different environmental factors. Subjects in the study were at least 70 years old at the time of their participation, and as mentioned the purpose was to investigate whether moderate alcohol consumption exerted a *causal* effect on cognitive functioning.

When moderate alcohol consumption was initially correlated with cognitive performance, alcohol consumption seemed to protect cognitive functioning. This would be a common endpoint of most observational research. Because of the twin component, however, it was possible to then estimate a series of twin-based mixed-level regression equations. These equations examined the impact of “differences” between the cognitive functioning of siblings based on differences in their drinking habits. For discordant DZ twins, the same finding as before emerged. The restriction of the model to MZ pairs, in an effort to more tightly control familial confounding (both environmental and heritable), revealed something different. No MZ discordance emerged for the outcome variable, suggesting that no causal effect was present. Speaking more fully to the insights provided by the models, McGue et al. ([2]; p.553) noted:

“*Given that we do not observe an association within MZ pairs, neither* *reverse causation (i.e., cognitive ability causing drinking) nor the contribution of unmeasured confounders underlying differences in exposure (e.g., nondrinkers are more likely than drinkers to be in poor health) seem to provide alternative explanations* *for our findings.*”

We mention the study by McGue and colleagues [2] in order to provide an initial example of an existing data sources available to study aging twins. The focus was on alcohol in this particular analysis, but it could have easily centered on a number of other important and interesting variables. The topic is less important, in this case, compared to the methods used as part of an attempt to create better causal inference capabilities when analyzing observational data.

The second example moves iteratively closer in the direction of using twins to study abuse, neglect, and other varieties of maltreatment in aging populations. Intimate partner violence (IPV) is a topic of longstanding interest to criminological and psychological researchers [34–36]. Aside from some limited ability to examine IPV within the context of experimental design [35, 37], it represents a topic that is often difficult to study via randomized trials (see also, [38]). What is interesting, though, is that while not many of them exist, there are a limited number of twin and sibling-based studies that have begun examining the topic.

Hines and Saudino [39] provided one of the first studies on the topic using twin data in a modestly sized sample of just under 200 MZ and DZ American twins. Barnes, TenEyck, Boutwell, and Beaver [40] followed some years later with a similar analysis in a large independent sample of twins from the United States (approximately 1,100 MZ and DZ twins). Both studies utilized straightforward variance decomposition models for different measures of IPV, and both arrived at roughly similar substantive conclusions ([41]; we are limited here by space, but for further discussion see [40, 42, 43]).^1^,^2^ Recalling McGue et al. [2], the most important point for this discussion is that one can imagine future research where potential protective factors and risk factors could both be modelled similar to alcohol consumption in order to examine various causal pathways leading not only to IPV, but to other measures of victimization as adults reached advanced ages.

Our final example here is somewhat more afield, but still seems relevant given the analytical approach employed. In order to provide a more granular analysis of the causal pathways related to maltreatment in childhood, Iob and colleagues [44] analyzed of over 200 participants in the Twins Early Development Study. Obviously the sample was comprised of children, not older adults, and one of the focal variables, “adverse childhood experiences” or ACEs, taps an array of deleterious experiences, up to, and including, emotional and physical abuse [45]. Both points should be kept in mind. It is the plan of analysis in the paper, however, that deserves focus here. Because the sample included twins it enabled Iob and colleagues [44] to test a plausible causal pathway spanning exposure to outcome. As the authors note (Iob et al., [44]; p.7):

“*Further, the mediation analysis indicated that cortisol mediated around 10–20% of the total associations of ACEs cumulative exposure, bullying, and dysfunctional parenting/emotional abuse with depressive symptoms. The relationships among ACEs, cortisol and depressive symptoms were generally attenuated when controlling for genetic liability, but both ACEs and cortisol remained as risk factors for later depressive symptoms.*”

If replicated, the potential translational value seems high, not unlike what might be achieved with similar strategies applied to samples of aging twins.

Prior to concluding, a couple of methodological points are worth contemplating. First, we make no assertion that the measurement, correlates, and causes of IPV (or ACEs) are necessarily going to be the same as those of abuse and neglect in older adults. A connected consideration, is that the retrospective nature of certain abuse measures, especially in younger individuals and including those used by Iob et al. [44], might strongly increase measurement error. Indeed, a path for error to creep in is when years accumulate between exposure and report [44]. With adult measures, one may limit some concerns about memory and recall given the opportunity to have more contemporaneous reporting periods. That said, measures of abuse and neglect in older adults will likely involve their own unique difficulties as well [5, 46].

The studies covered in this section are instructive because they demonstrate the overall plausibility of measuring outcomes of exposure to abusive and neglectful experiences in data comprised of siblings, as well as the capability of doing so at any stage of the life-course. Resources and time permitting, one existing and widely used dataset of aging twins in particular—The Vietnam Era Twin Study of Aging (VETSA)—might eventually collect such data from participating individuals, and may ultimately be the first to offer key insights on this front [47, 48]. The few studies mentioned, moreover, provide a reasonably good analytical roadmap for researchers interested in carrying out studies on the topic once data are more widely accessible.

### Maltreatment of older adults: from risk factors to causes and effects

While important work is ongoing concerning the maltreatment of older adults, there remains much left to do [49]. Starting first with the issue of prevalence, some national-level data suggests that approximately 10 percent of older adults experience some form of abuse annually in the United States [50], with somewhat higher rates emerging internationally [51]. By the year 2030, roughly 20 percent of the population could be over the age of 65, compared with the 14 percent above that threshold in 2012 [52], thereby increasing the number who might be victimized, either by a caregiver or family member.

The risks of victimization include a range of deleterious outcomes across the life-course [49, 53–55]. The challenge will continue to be carefully delineating *risk* factors from *causal* factors, while also mapping the various pathways running from causes to effects [see 44]). To help illustrate how possible research agendas might form around these goals, we focus on two well-known risk factors. A history of family violence, and dependency on others for care, represent two replicated risk factors which are primed for analysis with twin data [5].

Beginning with familial histories of violence, a point widely appreciated among scholars is that the effects of exposures can be heterogeneous for individuals, included cases when exposure happens across individuals in the same family [56]. In general, childhood victims experience an elevated risk of later perpetrating abuse against other family members later in life, particularly those who may have mistreated them in childhood [56, 57]. There is also an element of continuity which exists, given that older adults with histories of maltreatment continue to be at risk of further victimization as they age [58, 59]. As a more concrete example, we might imagine a scenario in which parents who abused their children start to incur increasingly high levels of risk for abuse themselves as they age, especially if they become physiologically frail rendering them dependent on others for care (a point we discuss more below).

Exacerbating this risk are the various health and cognitive impairments that humans eventually encounter as we age [60]. A specific study on the topic might examine the effects of differential exposure to different forms of maltreatment between siblings at relatively early stages of the life-course so as to then estimate their possible causal effects on health span outcomes at more advanced ages [44, 59, 61]. One could also test whether differential exposure to childhood (or adolescent) victimization, for instance, has causal influences on similar exposures in adulthood. This would take another meaningful step toward better illuminating casual pathways, in this case as they relate to continuity in risk. 

Dependency, our second example, flows naturally from the first and refers to an increased reliance on others for care (Cannell, Weitlauf, Garcia, Andresen, Margolis, & Manini, 2015). Individuals can experience increased in dependency at any age, yet the general consequence remains the same and includes an increase in the risk for various negative outcomes (Cannell, Weitlauf, Garcia, Andresen, Margolis, and Manini, 2015). With time, the risk of encountering one of the common diseases of aging, such as Alzheimer’s disease (AD), increases and can expedite a diminished ability to self-care [7, 46, 62]. Holding this example in mind, we can begin to describe various plausible pathways which might ultimately lead to abuse and neglect for aging individuals. A child previously exposed to maltreatment might eventually become involved in the care of an aging parent who perpetrated their victimization. Providing daily care to a loved one is challenging without a history of familial violence. When combined with such a history, it could make for situation primed for the abuse and neglect of older individual [5, 49, 56, 57, 63–68].

Indeed, psychological strain incurred by caregivers represents a variable highlighting for eventual inclusion in a twin-based study on the effects of dependency (see [5, 69–71]). As AD driven physical and cognitive deterioration increases a patient’s level of dependency, it might likewise be iteratively causing the likelihood of maltreatment or neglect to rise [7, 69–71]. Further compounding the problem is that abuse and neglect might go unrecognized or unreported for stretches of time owing to the declining functionality accompanying AD [5, 7, 70]. Another exposure variable, increased physical contact with a care-giver such as for hands-on lifting, might prove useful in future analyses. While not necessarily *the* primary causal factor for abuse, it seems to certainly increase the opportunities to experience harm and thus may represent a component of a causal chain [7].

Discussing dependency effects provides an opportunity to reiterate a point raised earlier. Maltreatment of older adults can manifest both as a *cause* of diminished health span as well as an *effect* of it [5, 7, 70]. We have attempted to include examples in this section that specifically illustrate this fact. A host of simple tasks, as they become increasingly challenging, can feed increases in dependency, which then might incrementally imperial the well-being of older adults. As levels of dependency rise, moreover, the location where care is being provided might enter the mix of factors that can act to steer risk in distinct ways, highlighting the importance of data drawn across a variety of settings in which older persons reside (see [72]).

This discussion of correlates is not exhaustive and is not intended to be. Rather, it returns us to the assertions guiding the arguments in our review. The first is that abuse and neglect of older adults should be considered fundamental in health span scholarship. The second is that quantitative genetic designs using twin data will be useful for this in the same way they have been essential to building knowledge across disciplines (for examples, see [73–76]). We included a small number of possible hypotheses and variables that might soon be examined using twin data, but in reality the list of interesting research questions is too long to list.^3^

Our arguments are coming to fruition already, at least to some extent, using data designed to study the aging process in samples of twins [2, 48]. By accelerating this trend, via the establishment of additional twin cohorts followed across advanced ages, health span science can build out a literature of causal effects related to variables which are virtually incapable of being studied experimentally. As the normal course of knowledge building proceeds, systematic reviews become possible, utilizing protocols such as PRISMA (http://www.prisma-statement.org/Protocols/) to highlight the findings which have emerged as most robust over time. Such approaches will doubtless deepen the reservoir of insight about how to live longer. Arguably more important, though, is the fact that they might also clarify how, in the course of being alive for more years, we might also enjoy a happier and safer existence.

## Data Availability

Not applicable.

## Footnotes

1 For these two studies in particular, different measures were used to assess IPV. Hines and Saudino [39] employed the psychological and physical aggression subscales of the Revised Conflicts Tactics Scale, which assesses both perpetration and victimization of IPV. Barnes and colleagues [40] utilized self-reported measures of physical aggression and violence perpetrated against one’s partner.

2 It seems worth mentioning that the purpose of using twin studies, and even calculating heritability estimates for something like IPV, is not equivalent to searching out some deterministic process in which individuals are destined from birth to be abusive toward loved ones. It also does not obfuscate the importance of external factors, such as culture. Studies designed in such a manner, in fact, help to control the nuisance factor of familial confounding, so that the importance of environmental, social and cultural factors can be illuminated and studied more clearly. See Hines and Saudino [39, 43, 44] for an excellent discussion.

3 Of course, this is not meant to imply that only twin studies should be used. Given the relative rarity of such studies, especially spanning birth to death, the field should, and must, continue to catalog evidence using a wide variety of sampling frames. One in particular concerns regulating care for older adults in nursing homes, given the widespread potential for abuse [72].
